# No Acute Effects of Choline Bitartrate Food Supplements on Memory in Healthy, Young, Human Adults

**DOI:** 10.1371/journal.pone.0157714

**Published:** 2016-06-24

**Authors:** D. P. Lippelt, S. van der Kint, K. van Herk, M. Naber

**Affiliations:** 1 Cognitive Psychology, Leiden University, Wassenaarseweg 52, Leiden, 2333AK, The Netherlands; 2 Leiden Institute of Brain and Cognition, LUMC, Leiden, 2300 RC, The Netherlands; 3 Experimental Psychology, Utrecht University, Heidelberglaan 2, Utrecht, 3584CS, The Netherlands; Waseda University, JAPAN

## Abstract

Choline is a dietary component and precursor of acetylcholine, a crucial neurotransmitter for memory-related brain functions. In two double-blind, placebo-controlled cross-over experiments, we investigated whether the food supplement choline bitartrate improved declarative memory and working memory in healthy, young students one to two hours after supplementation. In experiment 1, 28 participants performed a visuospatial working memory task. In experiment 2, 26 participants performed a declarative picture memorization task. In experiment 3, 40 participants performed a verbal working memory task in addition to the visuospatial working memory and declarative picture task. All tasks were conducted approximately 60 minutes after the ingestion of 2.0–2.5g of either choline bitartrate or placebo. We found that choline did not significantly enhance memory performance during any of the tasks. The null hypothesis that choline does not improve memory performance as compared to placebo was strongly supported by Bayesian statistics. These results are in contrast with animal studies suggesting that choline supplementation boosts memory performance and learning. We conclude that choline likely has no acute effects on cholinergic memory functions in healthy human participants.

## Introduction

Consuming sufficient nutrients is at the base of healthy physical functioning and mental well-being. The daily intake of dietary components is essential for the synthesis of the brain’s neurotransmitters and a lack of such chemical compounds can have serious consequences for health and behavior [1, 2]. One such dietary components is choline, which is believed to be important for healthy brain functioning and behavior.

Animal studies have shown that choline is a precursor of the major neurotransmitter acetylcholine [2–7], a neurotransmitter crucial for cholinergic memory functioning [8–16]. Choline is chemically very similar to acetylcholine [17] and the synthesis of acetylcholine largely depends on dietary choline intake [18–21]. Thus, choline is important for the synthesis of acetylcholine in animals [5]. As a result, the long-term effects of dietary choline availability on the rodent’s brain and memory have been investigated extensively. For example, the brain’s acetylcholine concentrations increase after an enriched choline diet [22] and memorization of food locations improve after rats were administered with choline either prenatally [23–27], around birth [26, 28], or later in life [29, 30] (for a review, see [31]). Furthermore, in cognitively impaired mice prenatal choline supplementation also enhances performance in typical attention and memory-related tasks [23–25]. Yet other studies have demonstrated a link between a choline deficient diet and impaired acetylcholine synthesis [32], and impaired memory in adult animals [4, 33, 34]. In sum, the studies above suggest that there is a strong link between dietary choline intake and memory functions in animals.

Due to the promising findings in animal studies, scientists have postulated that abnormalities in choline availability and acetylcholine synthesis may give way to the development of memory disorders such as Alzheimer and Parkinson dementia in humans [35–37]. Several studies have tried to improve memory functioning in aged human patients suffering from impaired memory by increasing the amount of choline in the diet. However, the results were mixed. Lecithin and choline chloride, both substances containing the chemical structure of choline, do not successfully improve memory performance [35, 36, 38–41]. However, cytidine diphosphate choline (citicoline, CDP-choline) and choline alphoscerate (alpha-glyceryl-phosphoryl choline, alpha-GPC), both alternative compounds that contain choline, seem to be promising substances in the treatment of progressively declining memory functioning in human patients with dementia [42–45]. The discrepancy between the effects of these different substances may relate to the complexity of the digestive system. Nonetheless, it remains unclear why and which choline-containing substances improve memory. Here we extend this field of research by testing the effects on memory functioning of another substance called choline bitartrate.

Many of the studies discussed above manipulated choline availability in the diet. However, the more acute effects of choline intake on memory performance have thus far not been investigated in humans. Recent evidence suggest that choline has acute effects on the motor system, which, as memory does, depends on cholinergic brain networks [46]. In the current study we therefore investigated whether choline can affect performance on a variety of memory tasks an hour after supplementation.

Most aforementioned studies used cholinergic substances to improve memory performance in elderly patients with memory impairments. A recent study has further examined the effect of choline on visual and auditory memory performance in healthy human adults, either by itself or in combination with caffeine [47]. Although choline on its own did not lead to significant improvements in memory performance they did find that a combination of 2g of choline bitartrate and 25mg of caffeine significantly improved performance on both the visual and auditory memory task compared to a placebo group. In a different experiment a combination of 100mg of caffeine and 2g of choline also significantly improved performance on a backwards digit span test compared to either caffeine or choline on its own [48]. This suggests that choline bitartrate does have the ability to improve memory functioning in healthy human adults, although the exact mechanism remains unclear. To examine whether high age and memory improvements matter for choline to have effect, and to examine a larger target group than just patients, we additionally investigated the effects of choline in a population of young, healthy, human adults. In sum, the current study had two a priori goals: to investigate whether choline bitartrate, a salt version of choline, could improve memory performance in healthy individuals and to test what types of memory functions are improved by the ingestion of choline.

## Experiment 1 –Visuospatial Working Memory

### Materials and Methods

The first experiment was designed to test the effects of choline bitartrate versus placebo intake on visuospatial memory performance. We used a double-blind, placebo-controlled, randomized cross-over design with a counterbalanced order of conditions.

#### Participants

Thirty human individuals were recruited through the use of fliers and an online advertisement placed on a university website for ongoing studies. Two participants dropped out after session one. We eventually gathered data from 23 females and 5 males (age M = 19.50, SD = 2.05, range = 18–28; body mass index: M = 22.67, SD = 2.72, range = 17.6–29.8). All participants were right-handed students, received study credit or money for participation, gave informed written consent on paper before the experiment, and were debriefed after the experiment. Participants were required to be between 18 and 35 years old, to have normal or corrected-to-normal vision, no cardiac, hepatic, renal, neurological or psychiatric disorders, personal or family history of depression, migraine and no medication or drug use. All female participants were tested only on days that they were using hormonal contraception. Women who did not use hormonal contraceptives were therefore excluded from participation. In this way hormonal confounds were limited, allowing for proper comparison across subjects (for details, see [49]). In line with previous studies on the acute effects of choline on behavior [46, 49–51] and to control for choline uptake unaffected by other supplements, participants were restricted from drinking alcohol the day preceding the study and participants were not allowed to have breakfast, coffee, or cigarettes before the experiment (overnight fasting, only water and caffeine-free fruit tea without sugar allowed). Participants were naïve to the purpose of the experiment and told that we investigated the effects of vitamin C on cognition. To prevent that the students’ knowledge about food supplements would confound the results, the experiment was deceptive: participants were led to believe that they drank orange juice enriched with vitamin C. The experiments conformed to the ethical principles of the Declaration of Helsinki and were approved by the local ethical committee “Commissie Ethiek Psychologie” (Approval number: 5430110878 and 9406292569; For ethics guidelines concerning food supplements, see http://media.leidenuniv.nl/legacy/richtlijnen-toediening-voedingssupplementen.pdf; Leiden University, Institute for Psychological Research). There were no minors (participants younger than 18 years old) involved in the here described experiments.

#### Apparatus & materials

Depending on the session, participants were given 400ml of orange juice including 2g of dissolved choline bitartrate or 2g of microcrystalline cellulose. Choline bitartrate contains 41.1% choline by molecular weight (104 g/mol choline in 253 g/mol choline bitartrate; Chemical info from Pubchem, an open chemistry database), resulting in the administration of approximately 800mg of choline. Previous studies that have shown effects of choline on memory performance in dementia patients [42, 43] required their subjects to take 1200mg of choline alphoscerate a day (257g/mol), thus containing 485mg of choline a day. We therefore assumed a one-time dose of 800mg well exceeding these dosages would suffice to produce an acute effect on memory functioning in healthy young adults. The given amounts were still well below the established 3.5g recommended upper level of daily intake for adults [52] for safety purposes and to control for the fact that people typically ingest smaller amounts of choline throughout the day (adequate intake of choline is ~500mg; Dietary Reference Intakes, Institute of Medicine). The choline bitartrate consisted of a white powder of pure choline bitartrate ordered from www.bulkpowders.co.uk. Similarly, the microcrystalline cellulose placebo also consisted of a white powder with a similar fine-grained structure as the choline bitartrate. The choline bitartrate and microcrystalline cellulose were dissolved into the orange juice out of sight of the participant and it was not possible to distinguish the choline bitartrate and placebo containing orange juice by either sight, smell, texture, or taste.

Stimuli were generated on an Asus Vivobook laptop computer with Windows 8 operating system (Microsoft), using MatLab (Mathworks) and the Psychophysics toolbox extension [53]. The presentation monitor displayed 1366 by 768 pixels at a 60-Hz refresh rate. Screen size was 26cm in width and 17cm in height, and the participant’s viewing distance to the screen was approximately half a meter. We measured heart rate (HR) and systolic and diastolic blood pressure (SBP and DPB) at the non-dominant arm with an OSZ3 automatic digital electronic wrist blood pressure monitor (Welch Allyn). Mood and arousal were measured using a pen and paper on a 9 by 9 pleasure/valence x arousal grid [49, 54].

#### Stimuli and procedure

First the participant’s HR, SBP, DBP, and subjective mood. These assessments were repeated an hour later right before the memory experiment and again approximately 30 minutes later after the last task was completed. After the first assessment, participants ingested 400ml of orange juice containing either dissolved choline bitartrate or placebo. Fourteen participants took choline bitartrate in session 1 and placebo in session 2, while the order was reversed for the remaining 14 participants (random group assignment). The sessions were conducted on 2 separate days with approximately a week between sessions. Choline bitartrate and placebo doses were prepared in sealed tubes by author MN and handed over to the naïve experimenters. None of the participants was able to detect any difference in the way the orange juice tasted between sessions.

The behavioral experiments were carefully timed to ensure that choline was taken up into the participant’s system. In rats choline levels peak approximately 30 minutes after ingestion [55, 56], and acetylcholine levels significantly raises after approximately 40 minutes [56] and remains high for at least 90 minutes in rats [18]. In humans choline bitartrate increases choline blood plasma levels within 1 hour after ingestion [57, 58] and with brain concentrations peaking around 2 hours until at least up to 3 hours after ingestion [59, 60]. Choline’s effects on the cholinergic peripheral system peaks between 1 and 2 hours after ingestion [46]. For these reasons participants had to wait a full hour before conducting the behavioral tasks in each session to ensure that choline was taken up into the system. The duration between supplement ingestion and the memory tasks was set to 60 minutes, not varying more than a couple minutes across participants. Approximately half an hour after ingestion, participants were offered an apple or orange to prevent hunger (both of which are known to contain no choline). Sessions took approximately 1 hour and 30 minutes each to complete. Participants also performed a visuospatial motor task which was unrelated to the memory task. Results of this task are published in a different article [46].

**Fig 1:**
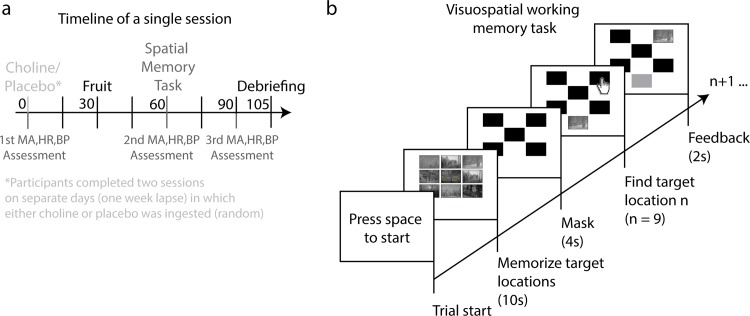
Timeline, procedure, and stimuli of the visuospatial working memory task of experiment 1. Participants received a 2 gram dose of either choline bitartrate or placebo in 2 separate sessions (cross-over design). A half hour after ingestion participants were allowed to eat some fruit and another half hour later they performed a visuospatial memory task (a). Mood & arousal, heart rate, and blood pressure (MA,HR,BP) were assessed 3 times during each session. The memory task was divided into 3 test blocks per session. In each block, participants completed 5 trials in which they had to memorize and consecutively indicate each of the location of the 9 target pictures. Pictures were shown for 10 seconds during which participants were supposed to memorize the locations of each picture (b). Pictures were then masked for 4 seconds and participants could subsequently indicate the location of the targets (random presentation order) with a computer mouse. Feedback was given by changing the target in a green (correct location) or red (incorrect location) patch. Pictures were shown in color but are here displayed in grayscale. The background color was grey during the experiment but is here displayed in white for aesthetical reasons.

The visuospatial memory task consisted of an adapted version of the popular children’s game “Memory” that is known to depend on cholinergic functioning in healthy human adults [61]. Participants played three blocks per session and the same pictures were presented in each block to facilitate learning. Each block consisted of 5 distinct trials (i.e., 5 distinct sets of 9 different pictures) in which participants had to memorize the location of 9 target pictures (150 by 225 pixels). The targets were simultaneously shown for 10 seconds in a 3-by-3 grid on a grey background (see Fig 1). To prevent that the task would be too easy and that participants would memorize simple features instead of entire pictures, the pictures were selected from a set of natural images [62] exclusively consisting of cluttered scenes of trees and plants and without individual objects and animals. Pictures were masked after presentation with a checkerboard pattern for 4 seconds. This was followed by a cue of the target picture that was shown below the grid. Participants had to retrieve the location of the target image from memory and click on its previous location with a computer mouse cursor. The image at the selected location then became visible for 2 seconds and the cue turned green or red depending on whether the participant had correctly or incorrectly indicated the location of the target, respectively. The next target was automatically shown following the feedback display and each of the 9 picture locations was consecutively tested within a trial. A total of 45 (5 x 9) pictures were tested within a block and each block lasted approximately 5 minutes. Participants were debriefed about the purpose of the experiment after the second session.

#### Statistical analysis

To test the effects of choline on working memory performance, we conducted a paired student samples t-test on the number of correctly memorized picture locations between the choline bitartrate and placebo sessions. HR, SBP, DBP, mood, and arousal were additionally tested for differences across sessions with the same analysis type. Bayes factors (BF) were calculated for the model “performance is larger after choline than placebo supplementation” in the statistics program JASP [63].

## Results & Discussion

**Fig 2:**
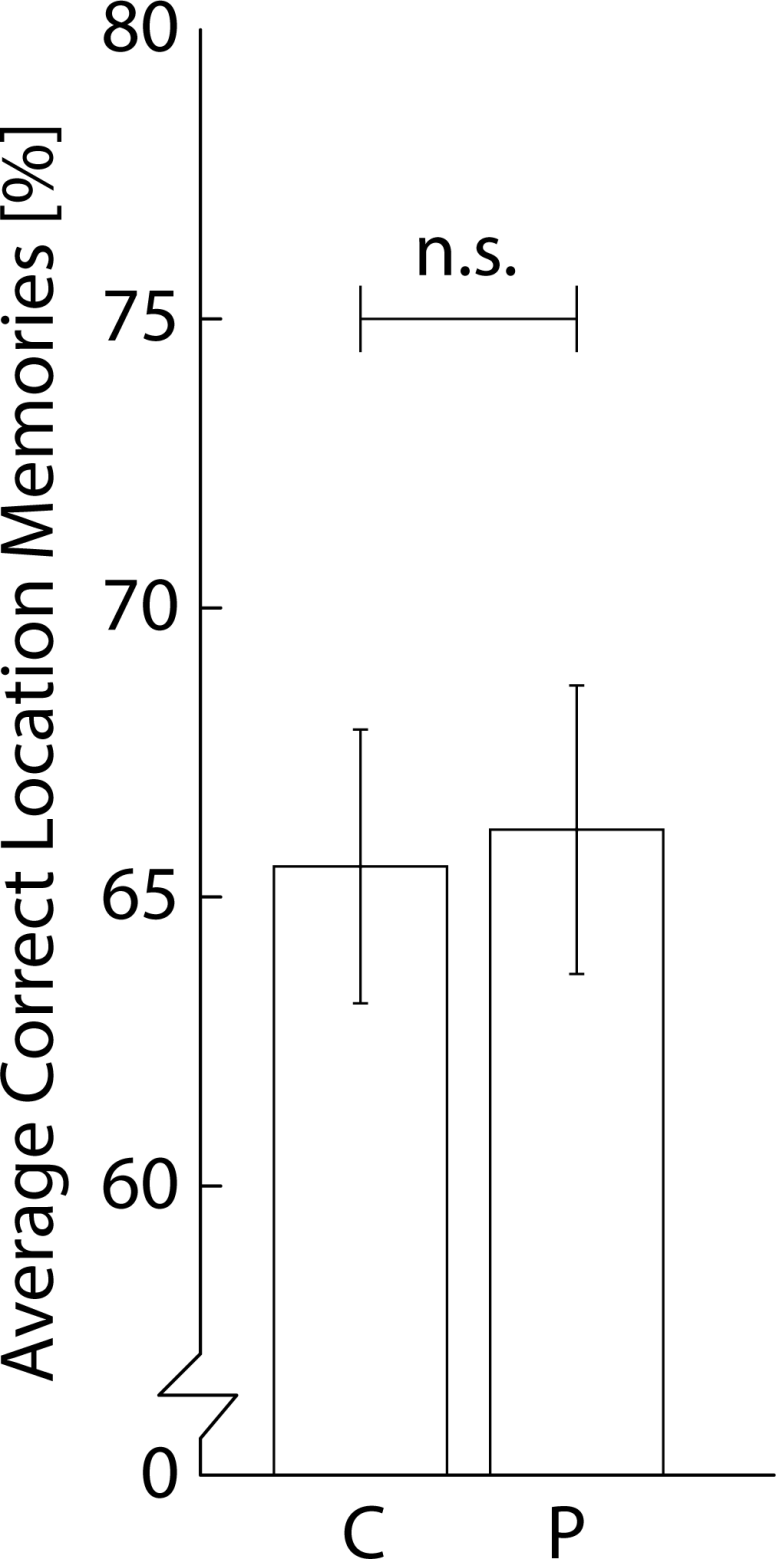
Results of visuospatial working memory task of experiment 1. Average percentage correctly memorized picture locations across participants (chance = ~11%) per supplementation condition (C = choline, P = placebo; n.s. = no significant difference).

Our first aim was to examine whether choline bitartrate improved visuospatial memory performance an hour after ingestion. A t-test showed no significant difference between the choline supplementation and placebo session on the number of correctly memorized picture locations (Fig 2; *t*(27) = 0.28, *p* = 0.780; Choline: M = 66%, SD = 13%; Placebo: M = 66%, SD = 13%). As the effect of choline may have depended on the participants’ Body Mass Indices (BMI), we additionally calculated the correlation between performance differences between choline and placebo and BMI. However, BMI did not correlate with the effect of choline versus placebo on performance (*r*(26) = 0.03, *p* = 0.899). Furthermore, we found no effects of choline on HR, SBP, DBP, mood, and arousal (*p’s* > 0.05; see S1 Table). Results obtained from the Bayesian analysis strongly favor the null-hypothesis that performance after choline supplementation is not better than after placebo supplementation (Choline > Placebo BF_10_: 0.164; BF_01_: 6.096). To summarize the results, choline bitartrate ingestion does not help healthy human participants to store and maintain locations of stimuli in working memory. In the following experiment we assessed whether declarative memories, probed through picture familiarity ratings, could be enhanced by choline bitartrate.

## Experiment 2 –Declarative Memory

In the second experiment we sought to assess choline’s effects on declarative memory instead of spatial working memory. Here we tested whether choline bitartrate intake improved recognition of pictures that had either already been shown in a preceding encoding phase (old stimuli) or had not been shown to the participants before (new stimuli). The study design, procedure, and apparatus were identical to experiment 1 except that the spatial memory task was replaced by the declarative memory task and we did not assess the participants’ subjective mood and arousal.

### Materials and Methods

#### Participants

Twenty-eight participants, naïve about the purpose of the experiment were tested in experiment two. The first participant was not able to complete the experiments due to technical problems and another participant did not show up for the second session. We ended up with data from 21 females and 5 males (age M = 21.18, SD = 3.49; range = 18–34; body mass index: M = 22.11, SD = 2.17, range = 19.0–26.8). Participants satisfied the same criteria as in experiment 1 but had not already taken part in experiment 1.

#### Stimuli and procedure

**Fig 3:**
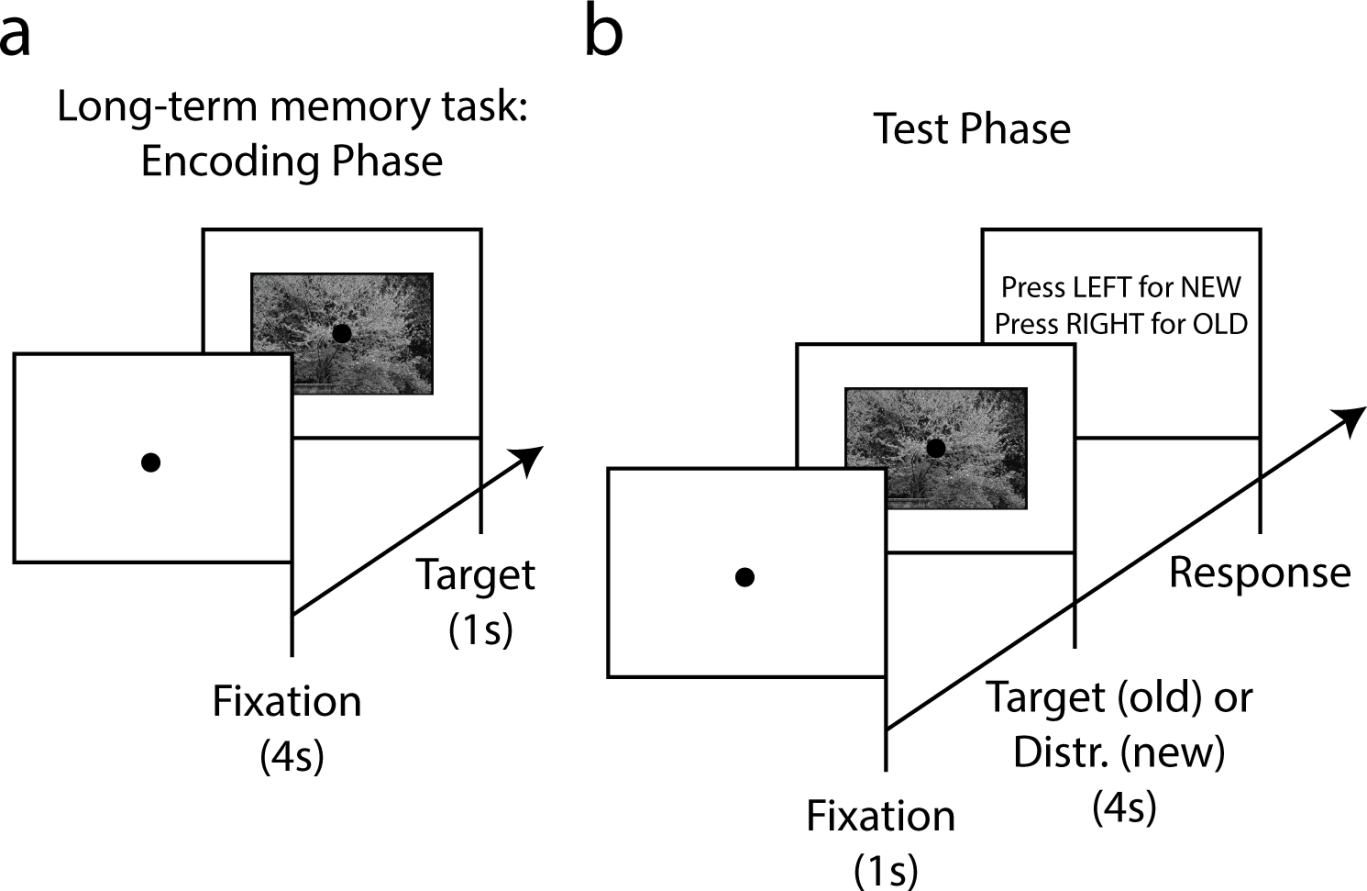
Procedure and stimuli of declarative memory task of experiment 2. Participants first memorized 50 pictures in an encoding phase (a). Next, the same pictures were presented intermixed with 50 new pictures (b). Participants indicated with 2 arrow buttons on a keyboard whether a presented picture was new (i.e., not presented during memorization) or old (i.e., previously presented).

Sixty minutes post ingestion, participants performed a declarative memory task which lasted approximately 30 minutes. The memory task was similar to the one described in Naber and colleagues [64]. First, participants had to memorize 50 target pictures (480 by 720 pixels) from the same database as used in experiment 1 (see encoding phase in Fig 3A). Each picture was shown for 1 second and preceded by a fixation dot for 4 seconds. The fixation dot was superimposed on the picture and participants were instructed to keep their gaze strictly on the dot. The encoding phase lasted approximately 4 minutes. A minute later following the encoding phase, the participants were tested on whether they had successfully encoded the images into declarative memory (the retrieval phase). The test images were shown intermixed with a new set of 50 pictures that were not previously shown in the encoding phase (Fig 3B). During the retrieval phase, participants viewed a fixation dot for 1 second, followed by the presentation of an old picture (i.e., previously shown) or a new picture for 4 seconds. The picture then disappeared and the participant was presented with the written instructions to press either the left or right arrow keyboard button to indicate whether the picture was new or old, respectively. The fixation point and subsequent picture were automatically shown after the response. There was no immediate feedback with regard to the accuracy of the participants’ response. The retrieval phase lasted approximately 10 minutes. Participants practiced the encoding and retrieval task for about 3 minutes with a different and easier set of 10 target images before the experiment.

#### Statistical analysis

Declarative memory performance was quantified by calculating the percentage of hit trials (old image recognized as familiar) and correct rejection trials (novel image recognized as unfamiliar). The same statistical tests as in experiment 1 were conducted.

## Results & Discussion

**Fig 4:**
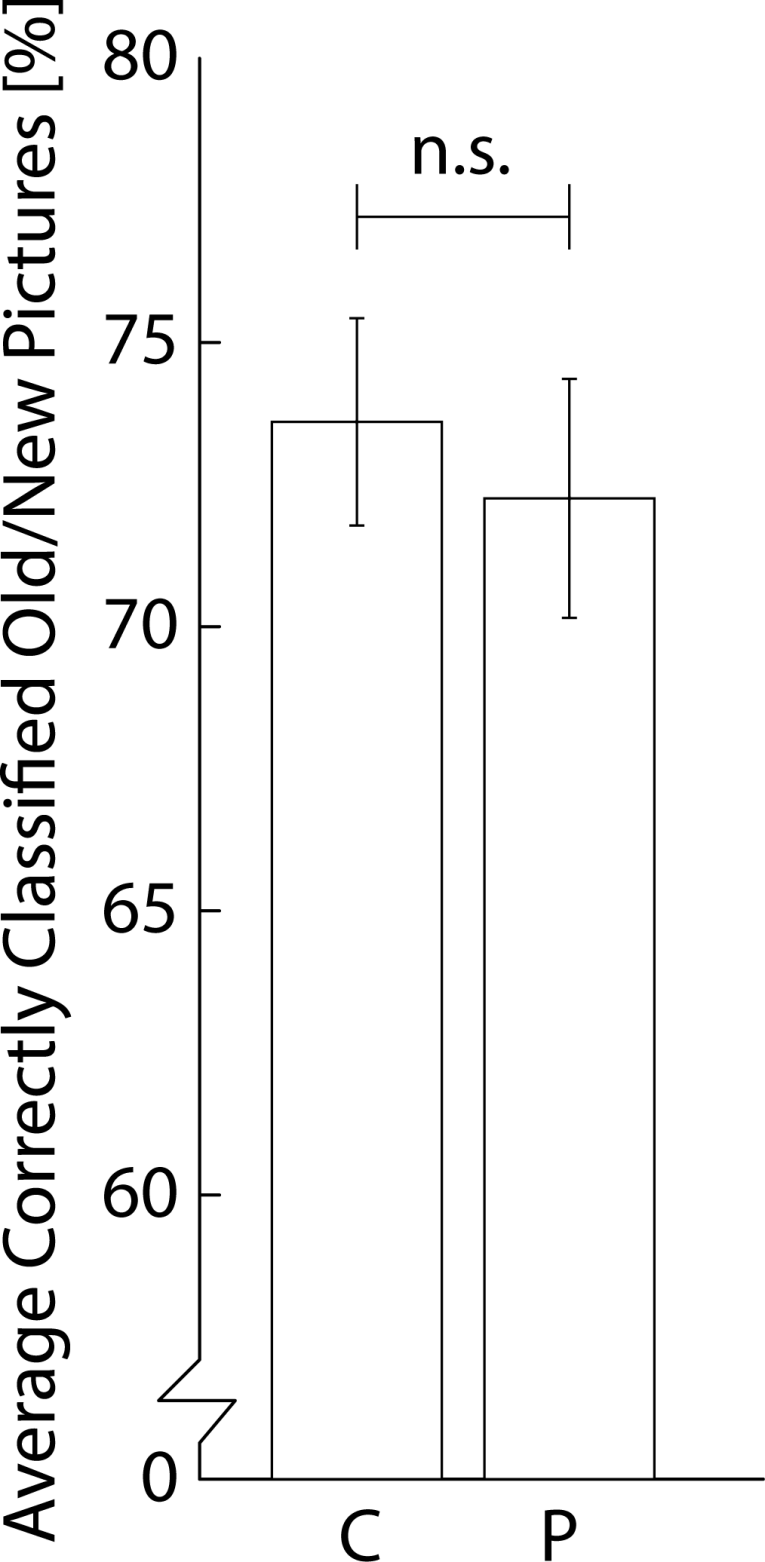
Results of declarative memory task of experiment 2. Average percentage of correctly classified pictures (chance = 50%) as old or new stimuli in the test phase across participants per choline and placebo condition.

We tested whether choline bitartrate improved declarative retrieval performance as compared to placebo and we found no significant effect (Fig 4; *t*(25) = 0.91, *p* = 0.372; Choline > Placebo BF_10_: 0.485; BF_01_: 2.063; Choline: M = 74%, SD = 9%; Placebo: M = 72%, SD = 11%). Body Mass Index did not correlate with the effect of choline bitartrate versus placebo on performance (*r*(26) = 0.14, *p* = 0.487). We found no effects of choline bitartrate on HR, SBP, and DBP (*p’s* > 0.05; see S2 Table). To conclude, the results showed that the retrieval of participant’s declarative memories were not improved by choline bitartrate supplementation.

## Experiment 3 –Replication and Verbal Working Memory

As the results from experiment 1 and 2 contrasted our expectations, we wondered whether the null-results were a consequence of low statistical power or low choline concentrations. Although the previously reported acute effects of choline bitartrate on visuomotor performance were highly significant and strong [46], its effects on memory could be more subtle. Hence, we designed a third experiment to replicate the results of experiment 1 and 2 but with a larger sample size, a larger stimulus set, and a higher dose of choline bitartrate supplementation. We also extended the set of tasks to assess choline’s effects on verbal working memory. Thus, the study design, procedure, and apparatus were identical to experiment 1 except that experiment 3 included an extended visuospatial working memory task, an extended declarative memory task, and an additional verbal working memory task.

### Materials and Methods

#### Participants

Forty participants were tested in experiment 3. Three participants did not complete the experiment for unknown reasons. We collected data from 30 females and 7 males (age M = 21.53, SD = 2.74; range = 18–29; body mass index: M = 22.05, SD = 2.80, range = 17.9–29.8). None of the participants had previously taken part in experiment 1 and 2.

#### Apparatus & materials

Depending on the session, participants were given 400ml orange juice including either 2.5g dissolved choline bitartrate or 2.5g dissolved microcrystalline cellulose. The choline bitartrate contained approximately 1g of effective choline. As the task included the presentation of auditory stimuli, participants wore head phones.

#### Stimuli and procedure

**Fig 5:**
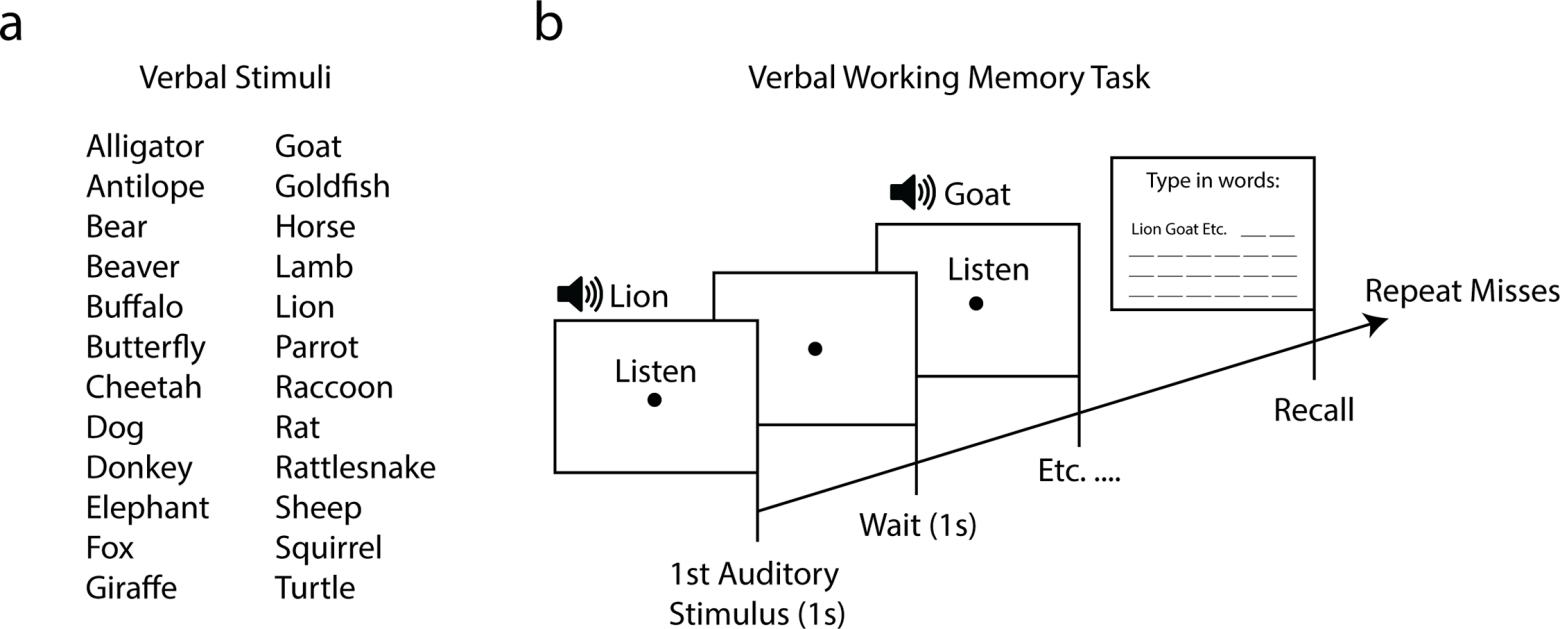
Procedure and stimuli of verbal working memory task of experiment 3. A total of 24 verbal animal species (a) were read out in 2 second intervals (b). Next, the participant had to recall and type in all the names they remembered. Unrecalled words (misses) were read out again until the participant was able to recall all words or until they had gone through 4 trial iterations.

The visuospatial working memory and declarative memory picture task were identical to the tasks used in experiment 1 and 2, except for the doubled amount of pictures. The verbal working memory task was a variant of the Buschke-task [65]. In the original Buschke task, the researcher reads out 24 names of animal species which are then to be recalled by the participant. Next, the researcher reads out those names that the participant was unable to recall and the participant is again asked to recall all of the 24 species names. This process is reiterated until the entire list of names is recalled correctly or until a fixed number of trial iterations has been performed. In the current version of the Buschke-task, the animal species names were read out by a female voice with a 2 second interval in which each auditory stimulus lasted approximately 1 second (Fig 5). Participants recalled the words by typing in their responses on the computer one-by-one. The maximum number of trial iterations was set to 5 (including the first trial). Because spelling could potentially pose a problem for some participants, alternatives such as “Cheeta” instead of “Cheetah” were also counted as correct recalls. Spelling errors were gathered during several pilot sessions.

#### Statistical analysis

Verbal working memory performance was quantified as the combined total of forgotten items across all trials.

## Results & Discussion

**Fig 6:**
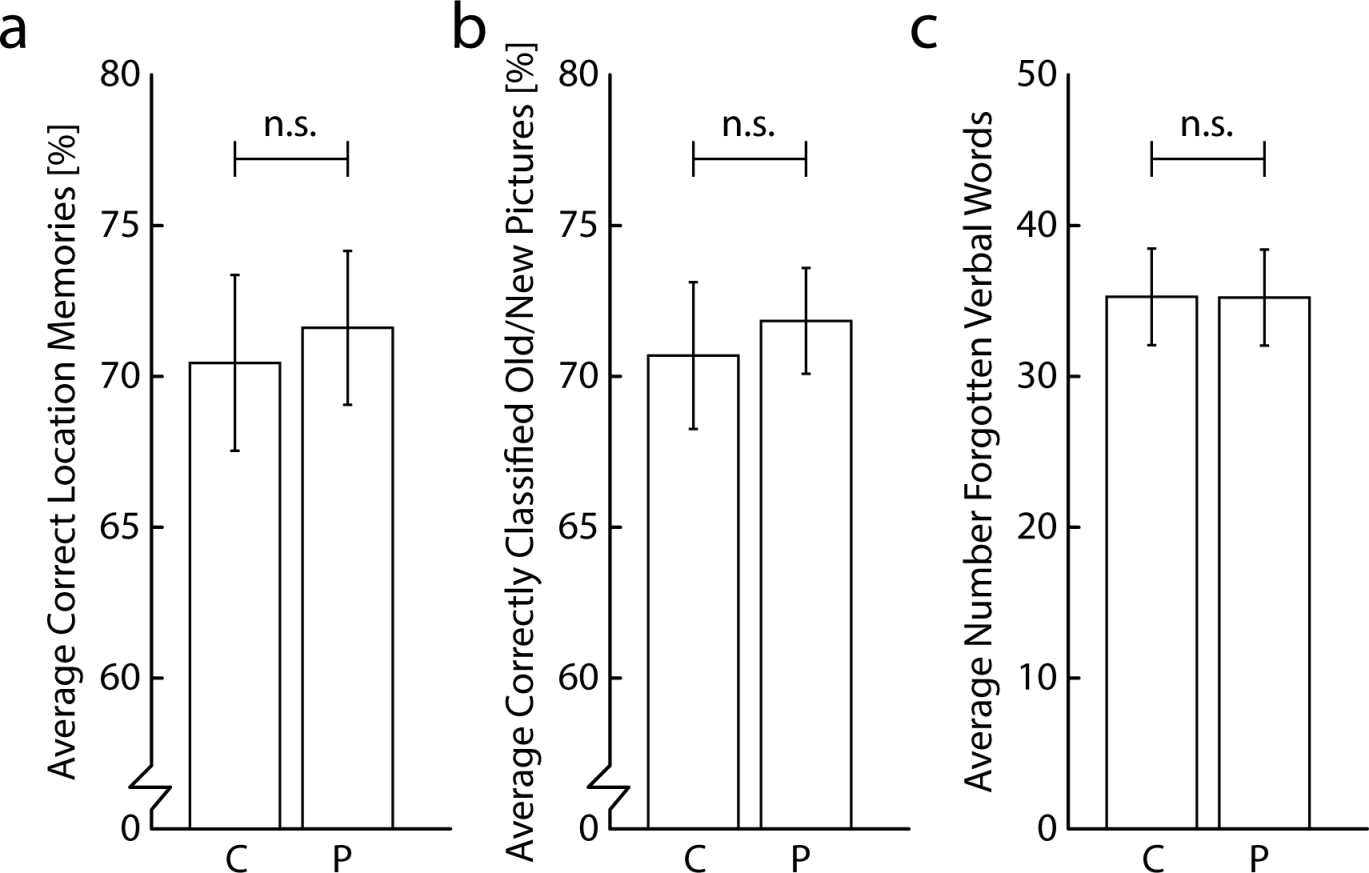
Results of memory tasks of experiment 3. Average percentage of correctly memorized picture locations in the visuospatial working memory task (a), percentage of correctly classified pictures as old or new stimuli in the declarative retrieval task (b), and number of forgotten items in the verbal working memory task (c) per choline and placebo condition.

Neither visuospatial working memory (*t*(36) = 0.49, *p* = 0.630; Choline > Placebo BF_10_: 0.268; BF_01_: 3.728; Choline: M = 70%, SD = 18%; Placebo: M = 72%, SD = 16%),), nor declarative retrieval (*t*(36) = 0.70, *p* = 0.489; Choline > Placebo BF_10_: 0.111; BF_01_: 8.963; Choline: M = 71%, SD = 15%; Placebo: M = 72%, SD = 11%), nor verbal working memory performance (*t*(36) = 0.02, *p* = 0.987; Choline > Placebo BF_10_: 0.179; BF_01_: 5.585; Choline: M = 35, SD = 20; Placebo: M = 35, SD = 19) were improved by choline bitartrate supplementation (Fig 6). BMI did not correlate with the effect of choline versus placebo for visuospatial working memory performance (*r*(35) = 0.10, *p* = 0.549), declarative retrieval performance (*r*(35) = 0.14, *p* = 0.487), and verbal working memory performance (*r*(35) = 0.02, *p* = 0.890). We found no effects of choline bitartrate supplementation on HR, SBP, DBP, mood, and arousal (*p*’s > 0.05; see S3 Table). To conclude, the results showed that memory functions, as measured with a variety of tasks and tested on a large sample of participants than in experiment 1 and 2, were not improved by choline bitartrate supplementation.

## General Discussion

In this study we have explored the effects of the dietary supplement choline bitartrate on memory functions in healthy human adults. Because choline is a precursor of the neurotransmitter acetylcholine, we expected positive effects on memory. However, no evidence was found of a beneficial effect of choline bitartrate supplementation on performance on a visuospatial working memory (experiment 1, 3), declarative long-term memory (experiment 2, 3), and verbal working memory task (experiment 3). These results suggest that choline bitartrate does not improve remembrance of spatial locations, declarative items, and verbal words.

Although these results are in contrast to expectations, there are a number of reasons that may explain the current findings. However, we first rule out a couple factors that could not underlie the null-results. The consistency of lacking differences between placebo and choline across all experiments deems it unlikely that statistical power was too low. Another possibility is that the effective dose of 1 gram choline may simply not be enough to have an effect. This, again, is not likely as similar amounts are known to affect behavior in humans [46]. The possibility that choline bitartrate was not synthesized to acetylcholine and could therefore not intervene with cholinergic functions is also unlikely. Choline is known to improve behavioral functions in healthy, young adults that rely on the cholinergic *peripheral* nervous system [46]. A perhaps more likely explanation is that the substance choline bitartrate may not affect cholinergic cell receptors in the *central* nervous system. Some chemicals may not be able to cross the blood-brain barrier but still affect the peripheral nerves. The blood-brain barrier actually keeps the central and peripheral neurotransmitter pools separate [66]. Alternatively, choline may not be synthesized into acetylcholine at the brain regions important for memory. This limitation would then have to be specific to choline bitartrate as the supplementation of other choline-containing substances result in rapid increases in acetylcholine levels a variety of brain regions in humans [57, 58, 60, 67, 68] and rats [22]. In line with this notion are results from a study showing that a single dose of 2g of choline bitartrate also did not produce any effects on either a visual or auditory memory task [47]. However, when combined with 25mg of caffeine performance was significantly improved on both tasks compared to a placebo group. The authors suggest that administration of choline bitartrate alone might lead to an oversaturation in choline availability unless the synthesis of acetylcholine is stimulated through increases in neuronal firing. Similarly, a combination of 100mg of caffeine and 2g of choline has been shown to significantly improve performance on a backwards digit span test compared to either caffeine or choline on its own [48]. Therefore the effectiveness of choline bitartrate on its own might be limited unless for instance combined with a substance such as caffeine which is thought to disinhibit neuronal firing through the release of adenosine. Another possible explanation is that we might have been unable to find an effect of choline bitartrate on memory performance due to individual differences in participants’ (baseline) neurochemical make-up. It is not uncommon for the relationship between the availability of a neurotransmitter and task performance to follow an inverted U-curve. Thus, additional acetylcholine synthesis might have both improved and impaired memory performance depending on a participant’s baseline position on the inverted U-curve. Lastly it might be possible that choline bitartrate has to be supplemented for a longer period of time before the memory system can benefit from its increased availability.

The interpretations outlined above are speculative and further scientific explorations are necessary before conclusions can be drawn on this issue. Unfortunately, it is not yet possible to directly measure choline-to-acetylcholine synthesis in the human brain and assess choline’s impact on cholinergic neural network activity.

The present study is the first to assess the acute effects of choline bitartrate supplementation on performance in a variety of memory tasks. Previous results on the effects of cholinergic substances on memory performance were mixed [35, 36, 38–45], possibly due to differences in populations and the type of cholinergic precursor. Future studies should therefore take into account the type of cholinergic substance used in the experiment. Another limitation of this study is the relatively large sample of female participants. It is first necessary to examine the effects of choline on a larger sample of males before the data can be generalized.

To this end, it would be interesting to study potential interactions across food supplements. Antioxidant, flavonoid, glucose, and fatty acid ingestion improve memory functions [69–75] and the administration of a cocktail including these supplements together with choline may be an interesting design for future studies. Future research may also further explore the role of choline in the peripheral nervous system as it is known to acutely affect pupil size after ingestion [46].

As a final remark, the data reported in this article question the impact of choline on memory functions in humans but also request for additional investigations to shed light on the actual mechanism behind choline supplementation and previously reported memory improvements. Nonetheless, we do not exclude the possibility that choline bitartrate could be an enhancer of other, yet untested cognitive skills. Nevertheless, while recent studies show that choline supplementation may help the elderly to recover from memory impairments [42–45], our experiments suggest that there are no beneficial effects of choline bitartrate administration on memory functioning in healthy, young individuals.

## Supporting Information

S1 Table Statistical results of additional assessments in experiment 1. Means, standard deviations, and statistical t-test results of physiological and subjective mood/arousal assessments after choline or placebo supplementation in experiment 1.
(DOCX)

S2 Table Statistical results of additional assessments in experiment 2. Means, standard deviations, and statistical t-test results of physiological assessments after choline or placebo supplementation in experiment 2.
(DOCX)

S3 Table Statistical results of additional assessments in experiment 3. Means, standard deviations, and statistical t-test results of physiological and subjective mood/arousal assessments after choline or placebo supplementation in experiment 3.
(DOCX)
