# Supplementary material for: No Acute Effects of Choline Bitartrate Food Supplements on Memory in Healthy, Young, Human Adults
Source: PLoS One. 2016 Jun 24;11(6):e0157714. doi: 10.1371/journal.pone.0157714 (PMC4920398; doi:10.1371/journal.pone.0157714)
Supplement: S1 Table — Means, standard deviations, and statistical t-test results of physiological and subjective mood/arousal assessments after choline or placebo supplementation in experiment 1. (DOCX) [file pone.0157714.s001.docx]

Supporting Information S1 Table

| **Factor** | **Drug** | **Placebo** | **t-value (df=27)** | **p-value** |
| --- | --- | --- | --- | --- |
| Heart Rate | 72±8 | 70±8 | 1.75 | 0.092 |
| Systolic Blood Pressure | 116±9 | 116±11 | 0.15 | 0.882 |
| Diastolic Blood Pressure | 73±7 | 72±7 | 0.95 | 0.352 |
| Mood | 6.38±0.86 | 6.35±0.95 | 0.30 | 0.769 |
| Arousal | 5.51±0.80 | 5.31±1.20 | 1.30 | 0.204 |
