# Supplementary material for: No Acute Effects of Choline Bitartrate Food Supplements on Memory in Healthy, Young, Human Adults
Source: PLoS One. 2016 Jun 24;11(6):e0157714. doi: 10.1371/journal.pone.0157714 (PMC4920398; doi:10.1371/journal.pone.0157714)
Supplement: S2 Table — Means, standard deviations, and statistical t-test results of physiological assessments after choline or placebo supplementation in experiment 2. (DOCX) [file pone.0157714.s002.docx]

Supporting Information S2 Table

| **Factor** | **Drug** | **Placebo** | **t-value (df=27)** | **p-value** |
| --- | --- | --- | --- | --- |
| Heart Rate | 69±9 | 67±9 | 1.67 | 0.107 |
| Systolic Blood Pressure | 115±9 | 116±9 | 0.89 | 0.383 |
| Diastolic Blood Pressure | 69±7 | 71±6 | 1.17 | 0.253 |
