# Supplementary material for: No Acute Effects of Choline Bitartrate Food Supplements on Memory in Healthy, Young, Human Adults
Source: PLoS One. 2016 Jun 24;11(6):e0157714. doi: 10.1371/journal.pone.0157714 (PMC4920398; doi:10.1371/journal.pone.0157714)
Supplement: S3 Table — Means, standard deviations, and statistical t-test results of physiological and subjective mood/arousal assessments after choline or placebo supplementation in experiment 3. (DOCX) [file pone.0157714.s003.docx]

Supporting Information S3 Table

| **Factor** | **Drug** | **Placebo** | **t-value (df=27)** | **p-value** |
| --- | --- | --- | --- | --- |
| Heart Rate | 70±10 | 72±10 | 1.15 | 0.257 |
| Systolic Blood Pressure | 117±9 | 120±9 | 1.11 | 0.275 |
| Diastolic Blood Pressure | 71±8 | 71±7 | 0.14 | 0.886 |
| Mood | 6.46±1.09 | 6.48±1.00 | 0.14 | 0.889 |
| Arousal | 4.62±1.62 | 4.95±1.47 | 1.40 | 0.171 |
